# A data driven approach in less expensive robust transmitting coverage and power optimization

**DOI:** 10.1038/s41598-022-21490-z

**Published:** 2022-10-22

**Authors:** Amir Parnianifard, Shahid Mumtaz, Sushank Chaudhary, Muhammad Ali Imran, Lunchakorn Wuttisittikulkij

**Affiliations:** 1 Department of Electrical Engineering, Faculty of Engineering, Chulalongkorn University, Bangkok, 10330 Thailand; 2 Instituto de Telecomunicações, Campus Universitário de Santiago, 3810-193 Aveiro, Portugal; 3 School of Engineering, Nottingham Trent University, Nottingham, UK; 4 School of Engineering, University of Glasgow, Glasgow, G12 8QQ UK

**Keywords:** Electrical and electronic engineering, Computational science

## Abstract

This paper aims the development of a new reduced-cost algorithm for a multi-objective robust transmitter placement under uncertainty. Toward this end, we propose a new hybrid Kriging/Grey Wolf Optimizer (GWO) approach combined with robust design optimization to estimate the set of Pareto frontier by searching robustness as well as accuracy (lower objective function) in a design space. We consider minimization of the energy power consumption for transmitting as well as maximization of signal coverage in a multi-objective robust optimization model. The reliability of the model to control signal overlap for multiple transmitting antennas is also provided. To smooth computational cost, the proposed method instead of evaluating all receiver test points in each optimization iteration approximates signal coverages using Kriging interpolation to obtain optimal transmitter positions. The results demonstrate the utility and the efficiency of the proposed method in rendering the robust optimal design and analyzing the sensitivity of the transmitter placement problem under practically less-expensive computational efforts (350% and 320% less than computational time elapsed using standalone GWO and NSGAII respectively).

## Background of study and motivations

The antenna placement problem or cell planning problem involves locating and configuring infrastructure for cellular wireless networks. From candidate site locations, a set needs to be selected against the objectives related to issues such as financial cost and service provision^1^. Antenna placement in a multi-antenna platform involves a manual process that is challenging and time-consuming and may result in a sub-optimal placement, leading to inferior performance of communication systems. The search space becomes exponentially large concerning the number of antennas to be placed (|search space|= ${m}^{n}$, where $m$ is the number of allowable locations for each antenna, and $n$ is the number of antennas)^2^. The antenna placement problem is well-known to be NP-hard^3–5^. Its solution has been attempted using heuristic approaches such as evolutionary algorithms (e.g., Genetic algorithm (GA)^6^), or swarm intelligence algorithms (e.g., Ant Colony Optimization (ACO)^7^, Particle Swarm Optimization (PSO)^8^). Another new swarm intelligence algorithm is Grey Wolf Optimizer (GWO)^9^. However, the literature lacks studies involving new swarm intelligence optimizers such as GWO in solving the optimal transmitter placement problem for wireless coverage systems. Besides, considering uncertainty as a source of variability in the computational model can significantly increase the cost of estimating the statistical figures of merit such as the mean and the Standard Deviation (Std) of the system response. In such cases, the application of fast surrogate models (also called metamodel) such as polynomial regression, Kriging, Artificial Neural Network (ANN), or Radial Basis Function (RBF), integrated into robust design optimization flow, allows for maintaining low computational cost^10–14^.

This paper proposes a new hybrid approach that employs Kriging interpolation surrogates and GWO as a new swarm intelligence methodology. The presented technique is combined with a dual-surface design to obtain (robust) optimal positions of the base stations in the transmitter placement problem under uncertainty. This integration is developed to achieve the design robustness along with accuracy in an optimal allocation of the base station transmitting antennas at a reduced computational cost. Also, we are interested in analyzing the sensitivity of the optimization results, in particular, computing the confidence regions caused by the randomness without extra function evaluations, which is ensured by employing the samples that are already acquired for the initial training of the model.

### Related works

Since the definition of the so-called Frequency Assignment Problem (FAP), i.e., a design problem focusing solely on the optimal assignment of transmitting frequencies, the optimal design of wireless networks has received much attention^15^. Over the years, more and more generalized versions of the problem have been taken into consideration, eventually leading to the consideration of multi-decision wireless network design problem versions that jointly consider the setting of the position, power emission, frequency, and modulation scheme of transmitters, see^16,17^. A transmitter placement scheme relies on a certain propagation model to assess the quality of a given transmitter allocation. For all cellular network systems, a major design step is to select the locations for the base station transmitters and to set up the optimal configurations such that the coverage of the desired area with sufficiently strong radio signals is high, and the deployment costs are low^18^. Interactive or automatic approaches have been used to produce an optimized transmitter placement based on certain propagation models^19–22^. When choosing the propagation model, one often needs to achieve a balance between the computational cost and the prediction accuracy^23^. Practical solution methods mainly differ in the optimization objectives and the search algorithms. Some metaheuristic methods have been developed for solving the transmitting placement problem.

In^24^, the authors adopt the ACO approach to optimize the transmitter locations to maximize the average received power. However, the benchmarking demonstrates that almost the same antenna locations are obtained with PSO and GA, whereas ACO requires more than ten times objective function evaluations (slow convergence), compared to PSO and GA. Like most evolutionary algorithms, GA has proven very capable of yielding high-performance antenna designs^2,25,26^. Determination of the base station position for optimum signal coverage using a particle swarm optimizer (PSO) has been investigated in^27^. The most important advantages of the GWO algorithm, compared to other optimization methods like GA, PSO, and ACO include (i) ease of implementation using fewer parameters for adjustment, (ii) owing to its simplicity and ease of implementation, GWO has gained significant attention and has been applied in solving many practical optimization problems since its invention^28^, (iii) the GWO algorithm has the convenient implementation and the advantages of not relying on parameter settings^29^, (iv) GWO has a high search efficiency, and the past seven years since it was first introduced^9^, have witnessed its rapid application to Wireless Sensor Networks (WSN) fields^29–31^, with acceptable capability in finding optimal coverage solutions in WSN within reasonable computational time (fast convergence to global optima)^32^. Application of metaheuristics to solve single or multi-criteria wireless network design problems serve as outstanding examples of the work being done to apply exact methodologies (i.e., to ensure convergence to an optimal solution) and applied to real-world large-scale problems in wireless network design.

Though in engineering design practice, the model has been impacted by the majority of external and environmental uncertainty or noise components^33^, causing the true response to be far from optimum points with system variation. By limiting the impacts of variation without eradicating the sources because they are either too difficult to manage or too expensive to do so, robust design optimization is an engineering way to enhance the performance of a model^34^. The robustness strategy's primary goal is to determine the optimal amount of design factor setting for achieving a desired system’s response that is insensitive to uncertainty as a source of variability^13,35^. Some recent development in the field of robust optimization can be found in^36–39^. Besides, considerable effort has also been put into using stochastic programming and robust optimization approaches to address robust versions of wireless network design challenges, as seen in^40–43^. However, in the coverage optimization problem, estimating the statistical measures due to uncertainty (the main source of antenna parameter variability) can considerably increase the computational cost. Consequently, the development of a less expensive approach, which—at the same time—offers sufficient reliability in searching for robust optimum is a practical necessity from the perspective of real-world communication system design.

To smooth the computational cost due to uncertainty, an accelerated data-driven method namely surrogates has been suggested properly^44,45^. Kriging surrogate (also known as the Gaussian process) is one the well-known surrogate that has been applied for different types of engineering design problems^46–48^. In^49^, the Kriging surrogate is used for transmitter location optimization in both single-transmitter and three-transmitter cases. In^50^, a comparative study between Kriging and GA for optimal transmitter location in an indoor environment has been performed from the fields scattered in the environment. In^51^, the PSO integrated with another surrogate named Radial Basis Function (RBF) has been applied to obtain the optimal placement of multiple transmitters by maximizing the overall signal coverage in an objective function, and controlling the intersection of transmitters in a constraint. In recent work^52^, a surrogate-based evolutionary algorithm by proposing the Mahalanobis sampling surrogate model assisting Ant Lion Optimizer (ALO) method has been applied to compute optimal coverage in a single objective three-dimensional WSN model. This study aims to employ Kriging assisted GWO to interpolate the whole network zone for the robust optimal placement of transmitting antennas due to some main reasons namely (i) in the electromagnetic community, Kriging is a recognized exact interpolation that has been applied intensively^53–55^, (ii) it highly flexible due to its ability to employ various ranges of correlation functions e.g., Gaussian, exponential, spherical and spline^56^, (iii) the Kriging method is extremely flexible in capturing nonlinear behavior (e.g., due to uncertainty) because the correlation functions can be tuned by the sample data^11,57^, and (iv) Kriging can provide a measure of error or uncertainty of the estimated surface^57^. Yet, instead of Kriging, it is common to employ another reputable surrogate such as ANN as its fitting functionality has been confirmed^58^. However, studying the application of other alternatives e.g., ANN, RBF, polynomial chaos expansion, and polynomial regression, in the robust coverage optimization of wireless networks is out of the scope of the current work. Notably, the quality of surrogate construction strongly depends on the distribution of training points, sample size, and optimally adjustments of hyperparameters of the surrogate. Hyperparameter optimization along with control on a number of expensive simulations keeping in mind the model doesn't get overfitted has been performed in literature^59^. Even though stochastic learning theory is responsible for the development of Kriging, the bounds on the hyperparameters are typically fixed arbitrarily^57,60^. There are new studies in the literature has been proposed the trade-off between model accuracy and sample size, with optimization of surrogate’s hyperparameters for surrogate model development, see^61,62^. In the current work, the space-filling design using the grid sampling method is used to design a training sample set. This method covers the whole design space by permuting sample points in equal ranges^63^.

### Main contributions

The major contributions of this study can be summarized as follows:

- The development of a new hybrid Kriging/GWO approaches combined with robust design optimization to estimate the Pareto front for the two objectives: design robustness and its (nominal) accuracy. We also provide a sensitivity analysis of transmitting antenna placement under uncertainty.
- Conducting numerical studies concerning multi-objective constrained robust optimization through minimization of the power consumption required by signal transmission, and maximization of the signal coverage as the objective functions. The control over the signal overlap is treated as the design constraint.
- In cases with a large number of receiver test points, the computational cost of the optimization process is very high because of evaluating the intensity of a signal received in all receiver points. The method developed in this work does not require exhaustive receiver evaluations in each optimization iteration.
- Carrying out extensive numerical experiments demonstrating the efficiency and reliability of the proposed algorithm in yielding robust optimal placement of multiple transmitters.

The rest of this paper is organized as follows. “Problem statement” section provides the preliminaries required by the proposed algorithm including a definition of the free space propagation model and a formulation of the relevant multi-objective robust design optimization concepts applied in this study. The algorithmic framework of the proposed reduced-cost approach to solving and analyzing the considered optimization task is presented in “Proposed Algorithm” section. The verification examples for the robust optimal placement of multiple transmitting antennas under uncertainty using the proposed approach are presented in “Experimental benchmark problems” section. “Conclusion” section concludes the paper.

## Problem statement

### Free space propagation model

This paper adopts the free space propagation model, which is widely used in the studies of placement problems^25,64^. The free space propagation model assumes a transmit antenna and a receive antenna to be located in an otherwise empty environment. Neither absorbing obstacles nor reflecting surfaces are considered^65^. The characteristics of an antenna may also be described in terms of the performance of a radio or radar system of which it is a part^66^. It is necessary to distinguish between the case of one-way transmission, in which a given antenna serves for transmission or reception only, and the case of radar or two-way transmission, in which a single antenna performs both functions. In this study, we consider a transmitting antenna and a receiving antenna separated by a large distance ${d}_{r,t}$. For propagation distances ${d}_{r,t}$ much larger than the antenna size, the far field of the electromagnetic wave dominates all other components. That is, we are allowed to model the radiating antenna as a point source with negligible physical dimensions. In such case, the energy radiated by an omni-directional antenna is spread over the surface of a sphere. This allows us to analyze the effect of distance on the received signal power.

**Figure 1:**
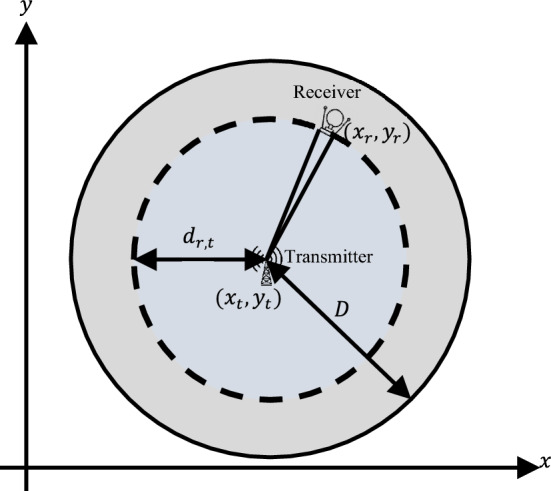
The free-space propagation model in a two-dimensional (2D) design space. Transmitting antenna is modelled as a point source. Transmitted power is spread over the surface area of a hypothetical sphere. The receiver antenna has an aperture. This surface with radius $D$ is called the “free-space coverage pattern for one-way transmission”, see Eq. (2).

Let ${G}_{t}$ and ${G}_{r}$ be the respective gain functions of the transmitting antenna and receiver antenna for the direction of transmission. In electromagnetics, an antenna's power gain or simply gain is a key performance number that combines the antenna's directivity and electrical efficiency. In a transmitting antenna, the gain describes how well the antenna converts input power into radio waves headed in a specified direction. In a receiving antenna, the gain describes how well the antenna converts radio waves arriving from a specified direction into electrical power. If the power transmitted is ${P}_{t}$, the power radiated in the direction of the receiver, per unit solid angle, would be $\left(\frac{1}{4\pi }\right){P}_{t}{G}_{t}$. If $\lambda$ is the carrier wavelength, the receiving antenna would present a receiving cross-section $\left(\frac{1}{4\pi }\right){G}_{r}\lambda$ to the incident wave; it would, in effect, subtend a solid angle $\frac{{G}_{r}{\lambda }^{2}}{4\pi {d}_{r,t}^{2}}$ at the transmitter. The power absorbed at the receiver would thus be^66^:

1 $${P}_{r} = \frac{{P}_{t}{G}_{t}{G}_{r}{\lambda }^{2} }{16{\pi }^{2}{d}_{r,t}^{2}}$$

where ${d}_{r,t}^{2}={\left\{{\left({x}_{r}-{x}_{t}\right)}^{2}+{\left({y}_{r}-{y}_{t}\right)}^{2}\right\}}$ stands for the Euclidean distance (2-norm) between locations of the receiver $r$ and the transmitter $t$ in a two-dimensional design space. The maximum operating range is determined by the signal-to-noise ratio of the detector system. If it is possible to ignore the effect of the earth on the propagation of the wave, and if ${G}_{r}$ is constant, it would be possible to operate the receiving system satisfactorily everywhere within the surface with a radius $D$ as below:

2 $$D = \left( {\frac{{{P_t}}}{{{P_{rm}}}}} \right)\frac{\lambda }{{4\pi }}{\left( {{G_t}{G_r}} \right)}^{{\raise0.7ex\hbox{$1$} \!\mathord{\left/ {\vphantom {1 2}}\right.\kern-\nulldelimiterspace} \!\lower0.7ex\hbox{$2$}}}$$

where ${P}_{rm}$ is the minimum detectable signal for the receiver, and the surface with radius $D$ is called the “free-space coverage pattern for one-way transmission”, see Fig. 1.

### Transmitter placement planning model

The planning model describes the environment and the mathematical model of the transmitter placement problem. The map for transmitter placement has two regions in a two-dimensional area ($X,Y$) including covered regions ($CR$) and placement regions ($PR$). The former shows the regions that need to be covered by a signal transmitted by antennas, and the latter represents the regions where the transmitters can be located. In this study, we consider the same free-space two-dimensional map (${\mathbb{Z}}^{2}$) region for both covered and placement regions with grid resolution $\delta$ where $CR\subseteq {\mathbb{Z}}^{2}$, and $PR\subseteq {\mathbb{Z}}^{2}$. In the placement planning model, a receiver gathers signals from the transmitters. The connectivity is assessed by a signal threshold $\theta$ to maintain the quality of service. This paper uses a large set $R$ of receivers as test points for the coverage: a receiver $r\in R$ has a position (${x}_{r},{y}_{r}$) $\in CR$ with threshold ${\theta }_{r}$. Let $EP$ be the set of energy power that can be transmitted by each transmitter, then the transmitter placement problem is to create a set of transmitters $T=\{t =({x}_{t},{y}_{t},{p}_{t})| {x}_{t},{y}_{t}\in PR, { p}_{t}\in EP\}$ and place its elements (i.e., the transmitters). In the current study, the transmitter placement problem is performed based on two objectives including maximization of percent coverage and minimization of Total Power Transmitted (TPT), when keeping the amount of coverage overlap (signal interference) under the predefined threshold. The latter is implemented as a design constraint.

*Percent coverage* A receiver $r\in R$ is said to be covered by a transmitter $t\in T$ when the signal strength is greater than the threshold, i.e.,

3 $$\mathrm{Covered}\left(r\right) = \left\{\begin{aligned} & 1,\quad \exists t\in T, {P}_{r}\ge {\theta }_{r}\\&0, \quad Otherwise\end{aligned}\right.$$

where ${P}_{r}$ is computed using Eq. (1). The value $1$ indicates that the receiver $r$ is covered by at least one transmitter. Accordingly, the percent signal coverage of a set of transmitters can be calculated by:

4 $$\mathrm{\%Coverage} = 100.\left(\frac{\sum_{r\in R}\mathrm{Covered}\left(r\right)}{\left|R\right|}\right)$$

Note that the first objective is to maximize the percent coverage.

*Total power transmitted* The proper planning model needs to be designed to minimize the total power consumed for transmitting signals by all designed transmitters while achieving as high percent coverage as possible. Thus, in this paper, apart from maximizing the percent coverage (see Eq. (4)), we consider minimizing the total power transmitted, i.e.,

5 $$\mathrm{TPT}=\sum_{t\in T}{p}_{t} ,\quad {p}_{t}\in EP$$

where ${p}_{t}$ is the power ($mW$) that is employed in transmitter $t$ for transmitting the signal.

*Overlap* The coverage overlap between transmitters raises the issue of interference^18,25^. To reduce the interference, we add a constraint to the model to keep the overlap under the predefined threshold. The relevant mathematical formulation of the overlap and the associated constraint will be explained in the next section.

## Proposed algorithm

### Materials and methods

#### Kriging

Kriging is an interpolation method that can cover deterministic data and is highly flexible due to its ability to employ various ranges of correlation functions^57^. In a Kriging model, a combination of a polynomial model and the realization of a stationary point is assumed by the form of:

6 $$y=f\left(X\right)+Z\left(X\right)+\varepsilon$$

7 $$f\left(X\right)=\sum_{p=0}^{k}{\widehat{\beta }}_{p}{f}_{p}\left(X\right)$$

where the polynomial terms of ${f}_{p}\left(X\right)$ are typically the first or the second-order response surface approach and coefficients ${\widehat{\beta }}_{p}$ are regression parameters ($p=\mathrm{0,1},\dots ,k$). This type of GP approximation is called the universal GP, while in the ordinary GP, instead of $f\left(X\right),$ the constant mean $\mu =E\left(y\left(x\right)\right)$ is used. The term $\varepsilon$ describes the approximation error and the term $Z\left(X\right)$ represents the realization of a stochastic process which in general is a normally distributed Gaussian random process with zero mean and variance ${\sigma }^{2}$, and non-zero covariance. The correlation function of $Z\left(X\right)$ is defined by:

8 $$Cov\left[Z\left({x}_{k}\right),Z\left({x}_{j}\right)\right]={\sigma }^{2}R\left({x}_{k},{x}_{j}\right)$$

where ${\sigma }^{2}$ is the process variance and $R\left({x}_{k},{x}_{j}\right)$ is the correlation function that can be chosen from different correlation functions which were proposed in the literature (e.g. exponential, Gaussian, linear, spherical, cubic, and spline)^67,68^.

#### Grey wolf optimizer

The canonical GWO is one of the recently proposed swarm intelligence-based algorithms, which is developed by Mirjajili et al.^9^ in 2014. It has been widely tailored for a wide variety of optimization problems due to its impressive characteristics over other swarm intelligence methods: it has very few parameters, and no derivation information is required in the initial search. The GWO has recently gained a very big research interest with tremendous audiences from several domains in a very short time^28,32^. It mimics the social leadership and hunting behavior of grey wolves. In the GWO algorithm, the fittest solution in the population is named alpha ($\alpha$). The second and third best solutions are called beta ($\beta$) and delta ($\delta$), respectively. The rest of the individuals in the population are assumed as omega ($\omega$). Grey wolves encircle prey during the hunt. In order to mathematically model encircling behavior the following equation is proposed^9^:

9 $$\overrightarrow{X}\left(t+1\right)={\overrightarrow{X}}_{p}\left(t\right)-\overrightarrow{A}.\left|\overrightarrow{C}.{\overrightarrow{X}}_{p}\left(t\right)-\overrightarrow{X}\left(t\right)\right|$$

where $t$ indicates the current iteration, $\overrightarrow{A}$ and $\overrightarrow{C}$ are coefficient vectors, ${\overrightarrow{X}}_{p}$ is the position vector of the prey, and $\overrightarrow{X}$ indicates the position vector of a grey wolf. The vectors $\overrightarrow{A}$ and $\overrightarrow{C}$ are calculated as follows:

10 $$\overrightarrow{A}=2\overrightarrow{a}.{\overrightarrow{r}}_{1}-\overrightarrow{a} \,\,\text{and}\,\, \overrightarrow{C}=2{\overrightarrow{r}}_{2}$$

where ${r}_{1}$ and ${r}_{2}$ are random vectors in $\left[0, 1\right]$, and components of $\overrightarrow{a}$ are linearly decreased from $2$ to $0$ throughout iterations by equation $\overrightarrow{a}\left(t\right)=2-\frac{2t}{MaxIter}$ where $MaxIter$ indicates the total number of iterations. The other wolves update their positions according to the positions of $\alpha$, $\beta$, and $\delta$ as follow^9^:

11 $$\overrightarrow{X}\left(t+1\right)=\frac{1}{3}\left({\overrightarrow{X}}_{\alpha }\left(t\right)-{\overrightarrow{A}}_{1}.\left|{\overrightarrow{C}}_{1}.{\overrightarrow{X}}_{\alpha }\left(t\right)-\overrightarrow{X}\left(t\right)\right|+{\overrightarrow{X}}_{\beta }\left(t\right)-{\overrightarrow{A}}_{2}.\left|{\overrightarrow{C}}_{2}.{\overrightarrow{X}}_{\beta }\left(t\right)-\overrightarrow{X}\left(t\right)\right|+{\overrightarrow{X}}_{\delta }\left(t\right)-{\overrightarrow{A}}_{3}.\left|{\overrightarrow{C}}_{3}.{\overrightarrow{X}}_{\delta }\left(t\right)-\overrightarrow{X}\left(t\right)\right|\right)$$

where ${\overrightarrow{A}}_{.}$ and ${\overrightarrow{C}}_{.}$ are obtained by relevant expressions in Eq. (10). The pseudo-code of the GWO algorithm is presented in Algorithm 1.
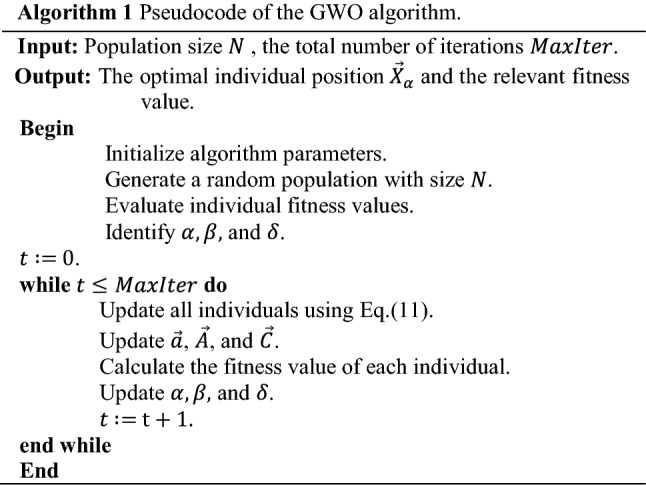


#### Robust dual-surface design

**Figure 2:**
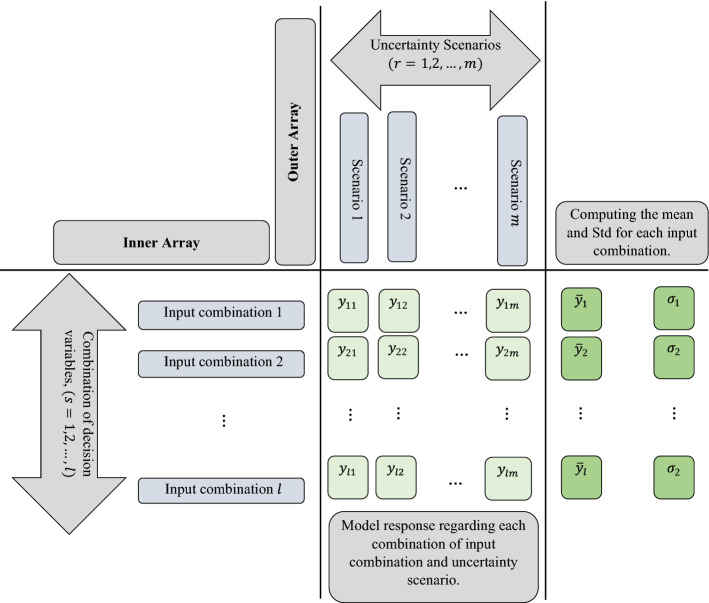
Visualization of crossing the model design parameters and uncertainty scenarios as inspired by the Taguchi’s crossed array design.

The dual response surface approach has been successfully applied in robust process optimization^69^. There are different robust optimization methods in the class of dual response that has been developed in the literature, see^14,69,70^. Here, we follow^71,72^ and inspire Taguchi’s overview of robust design^73^ for dealing with uncertainty as a source of variability in the model. However, we expand Taguchi's robust design terminology and apply its definition for environmental noise factors in such a multiple transmitters system under uncertainty. But in this study, we replace the statistical approach of the Taguchi viewpoint with hybrid Kriging and GWO approach. Furthermore, we intersect two experimental designs (data sample sets). The first design is pertinent to decision variables (inner array), whereas the second one is for uncertain variables (outer array). Given the vector $s=\left(\mathrm{1,2},\ldots , l\right)$ of sample points over the decision variables, and the vector $r=\left(\mathrm{1,2},\ldots , m\right)$ of uncertainty scenarios, $l\times m$ input combinations are designed, and the true model is evaluated $l\times m$ times to collect relevant model responses, see Fig. 2. Let $Y$ be the $l\times m$ matrix of model responses. The mean and the standard deviation (Std) for each row of $Y$ can be computed as

12 $${Mean}_{s}=\frac{1}{m}\sum_{r=1}^{m}{ y}_{sr} ,\quad\,\,for\, \,\left(s=\mathrm{1,2}, \dots , l\right)$$

13 $${Std}_{s} =\sqrt{\frac{1}{m}\sum_{r=1}^{m}{y}_{sr}^{2}- {\left(\frac{1}{m}\sum_{r=1}^{m}{ y}_{sr}\right)}^{2}} ,\,\,\,for \, \left(s=\mathrm{1,2}, \dots , l\right)$$

The following Signal-to-Noise Ratio (SNR) as a robustness criterion can be formulated:

14 $${SNR}=10\mathrm{log}\left[{Mean}^{2}+{\omega {*Std}}^{2}\right]$$

Since the goal is to minimize the model response, the formulation of the SNR in (14) has the opposite sign of the Taguchi formulation^73^.

### Mathematical optimization model

Numerical optimization uses a compact mathematical model for describing the problem of concern. Here, we define the problem of multiple transmitting antenna placement under uncertainty in the framework of robust multi-objective optimization. The goal is to obtain the set of transmitters’ optimal positions on a 2D map and the relevant optimal power for each positioned transmitter, $\left\{{t}^{*} =\left({x}_{t}^{*},{y}_{t}^{*},{p}_{t}^{*}\right) |{t}^{*}\in T\right\}$ in $T=\{t =({x}_{t},{y}_{t},{p}_{t})| {x}_{t},{y}_{t}\in PR, { p}_{t}\in EP\}$ where $PR\subseteq {\mathbb{Z}}^{2}$ determines allowable placement regions, whereas $EP$ is the set of power that can be transmitted by each transmitter. The objectives and constraints are defined as below:

15 $$\begin{aligned} &\,\,\,\quad\quad\quad Minimize SNR_{ } \hfill \\ &\quad\quad\quad\,\,\,Minimize TPT \hfill \\ & {\text{Subject to}}: \hfill \\ &\quad\quad\quad\quad\,\,\,prob. \left( {overlap} \right) \ge 1 - \beta \hfill \\ \end{aligned}$$

The first objective is constructed to make a trade-off between the mean and Std of signal coverage in the model. The SNR criterion is defined by

16 $${SNR}_{ }=10\, \mathrm{log}\left[{{\left(\frac{1}{\mathrm{\%}Coverag{e}_{Mean}}\right)}_{ }^{ }}^{2}+ {\omega .\left(\mathrm{\%}Coverag{e}_{Std}\right)}^{2}\right]$$

In Eq. (16), the terms $\mathrm{\%}Coverag{e}_{Mean}$ and $\mathrm{\%}Coverag{e}_{Std}$ denote the mean and the standard deviation of the coverage, estimated based on the variability of uncertain parameters in the model, using Eqs. (12) and (13). This objective is formulated to enable maximization of the percent coverage as well as robustness.

The second objective function considers the minimization of the total power (expressed in mW) consumed by all transmitters as follows

17 $$TPT=10\mathrm{log}\left[{\left(\frac{\sum_{t\in T}{p}_{t}}{Ntrans.{P}_{max}}\right)}^{2}+1\right]$$

The expersions $Ntrans$ and ${P}_{max}$ in Eq. (17) represent the number of transmitters in a model, and the maximum allowed power that can be allocated to each transmitter, respectively.

The probability of overlap (the intersection) between all transmitters that existed in the model is kept within the predefined threshold in the constraint of the model by

18 $$\begin{gathered} \frac{2}{{Ntrans.\left( {Ntrans - 1} \right)}} \hfill \\ \left[ {\mathop \sum \limits_{i \in T} \mathop \sum \limits_{j \in T, i \ne j} \Phi \left( {\frac{{d_{i,j} - \left( {Mean_{Di} + Std_{Di} } \right)}}{{SD_{i} + SD_{j} }}} \right)} \right] \ge 1 - \beta \hfill \\ \end{gathered}$$

The term $\Phi$ refers to the cumulative distribution function (CDF) of a standard normal distribution. The term $0\le \beta \le 1$ can be defined by the designer and represents the allowed probability for the average overlap of transmitters in the model. The expressions ${Mea{n}_{D}}_{.}$ and ${St{d}_{D}}_{.}$ represent the mean and the standard deviation of the radius $D$ (see Eq. (2)). In general, regarding each transmitter in the model, three design variables need to optimally be investigated using the proposed approach. The coordinate of each transmitter and transmitting power (design variables regarding each individual transmitter) need to be used to compute the percent coverage provided by each transmitter, see Eqs. (1), (2), and (3). This procedure is applied to all transmitters in the model. However, the overlap (intersection) between signal coverage by transmitters also is controlled by defining the probability of intersection that is kept under a predefined threshold in the models’ constraints (Eq. 18).

As both objective functions are expressed on the same logarithmic scale as $10\mathrm{log}\left(\varepsilon \right)$, where $\varepsilon \in \left[\mathrm{0,1}\right]$, we can aggregate them as

19 $$overall \,function=\frac{SNR}{1+{e}^{-\alpha }}+\frac{{e}^{-\alpha }.\left(TPT\right)}{1+{e}^{-\alpha }}$$

where $\alpha \in \left[-2,+2\right]$ is the weighting factor. The optimum selection of $\alpha$ depends on the designer’s preferences and the characteristics of the optimization model. Sweeping $\alpha$ allows for capturing the Pareto front, and for identifying trade-off designs between the considered objective functions^74^.

### Computational cost

One of the main difficulties in solving the above-mentioned mathematical model for multiple transmitters’ placement problems under uncertainty is obtaining the estimation of statistical measures including mean and Std of response due to variability of uncertain parameters. However, repetitions of the original model to estimate these statistical measures can increase the computational cost, while also may not provide accurate estimation in complex and non-linear models^75–77^. The main issue is computational cost due to the required large number of function evaluations. This paper uses a large set $R$ of receivers as test points for coverage: a receiver $r\in R$ has a position (${x}_{r},{y}_{r}$)$\in CR$ with threshold ${\theta }_{r}$. The grid resolution $\delta$ of the map and the threshold ${\theta }_{r}$ is the same for all receiver test points. We are interested to obtain the optimal positions and power for multiple transmitters in a 2D map ($X\times Y {\mathrm{m}}^{2}$). So, with resolution $\delta$, there is $[(X\times Y)/\delta ]$ possible base placements for each transmitter. In a stochastic model by considering the uncertainty, we also need $m$ repetitions of model to obtain the estimation of statistical measures including mean and Std of response due to variability of uncertain parameters. Notably, $m$ needs to be large enough to decrease the error of estimation, hence often, random sampling method for Monte Carlo-based uncertainty quantification has been applied^78–80^. In this paper, we apply the non-parametric space-filling design that applied a smaller number of sample points than the classical Monte Carlo method^81,82^. Let's assume $n$ is the number of transmitters that need to be optimally designed in a stochastic model (under uncertainty) and the standalone optimizer to investigate a robust optimal solution is adjusted by total $s$ individual runs (for instance for GWO the total number of optimizer’s runs is equal to “$Searc{h}_{Agent{s}_{No}}\times Ma{x}_{Iterations}$”), so the total number of required function evaluations is equal to [$n\times m\times s\times \frac{\left(X\times Y\right)}{\delta }]$. Thus, in a multi-transmitter placement problem particularly under uncertainty, directly applying metaheuristics such as evolutionary algorithm as used in^1,18,25,83^ or swarm-intelligence as applied in^24,27^ imposes high computational cost due to a large number of function evaluations. These techniques that require a large number of fitness evaluations to obtain robustness besides accuracy (lower objective function) are often limited to directly being applied to computationally expensive engineering problems under uncertainty, therefore, surrogate-assisted metaheuristic optimization algorithms have been proposed in the literature, see^84–86^.

### Algorithmic framework

**Figure 3:**
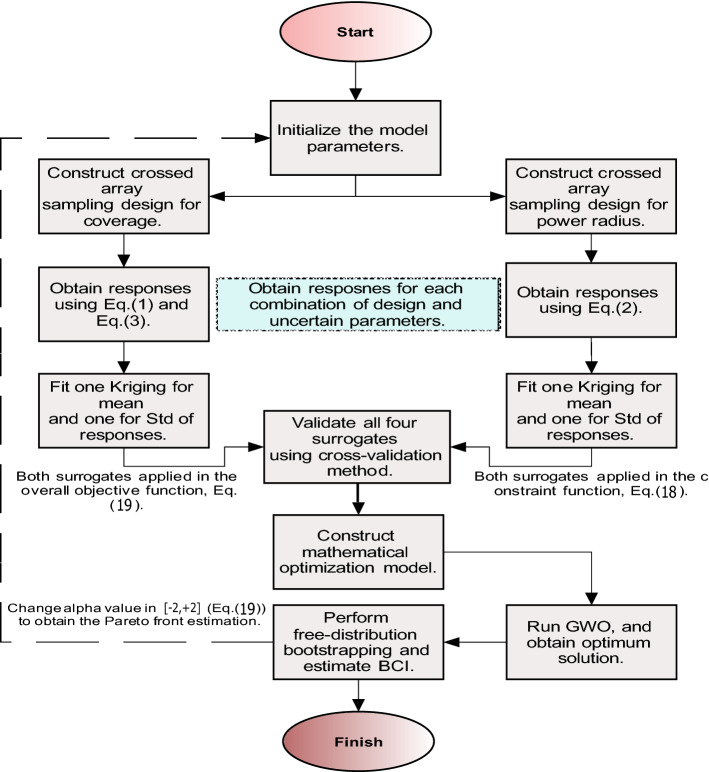
The proposed algorithm for multiple transmitter placement problem under uncertainty. In the proposed algorithm, two set of experiments are designed regarding the crossed array design, by setting decision variables in an inner array and uncertain variables in an outer array. Two surrogates are fitted, one over mean and another over Std of coverage. Another crossed array design constructed with the same framework for power radius.

Figure 3 provides the flow diagram of the proposed optimization approach. The algorithmic procedure of proposed hybrid approach in pseudocode is provided in Algorithm 2 and proceeds as main steps below:

- *Step 1* Initialize the model parameters.
  The model parameters and constants that need to be adjusted at the beginning of the algorithm are shown in Table 1. As can be seen, some parameters need to be adjusted initially by the decision-maker before running the optimization procedure (e.g., shown with “initialize”). Besides, we define three design variables including the transmitter coordinates $\left(x,y\right)$ in the 2D map and required power transmitted for each transmitter Furthermore, according to the number of transmitters in the model, the number of design variables is equal to $3\times No.transmitters$, while these variables are searched for optimal values during the optimization procedure. Note that in the current study, we consider the transmitting antenna gain (${\widetilde{G}}_{t}$) in Eq. (1) as an uncertain parameter that is uniformly varied in a predefined range with known lower and upper bounds. Among the proposed optimization procedure, we aim to reduce the sensitivity of optimal design variables (transmitter position in 2D map and required power transmitted) against this source of variability (uncertainty in transmitting antenna gain).

**Table 1:** The list of parameters applied in the proposed algorithm.

Parameter	Title	Unit	Value
${G}_{t}$	Transmitting antenna gain	mW	Uncertain variable (uniform distribution)
$\left({x}_{t},{y}_{t}\right)$	The transmitter coordinate in a 2D map	–	Design variables
${P}_{t}$	Power transmitted	mW	Design variable
$\delta$	Grid resolution	m	Initialize
${G}_{r}$	Receiving antenna gain	mW	Initialize
$\lambda$	Wavelength	m	Initialize
$X\times Y$	Two-dimensional map area	m^2^	Initialize
$\beta$	The allowable probability for average overlap of transmitters, Eq. (17)	–	Initialize
${\theta }_{r}$	Signal threshold in receiver point to maintain the quality of service	2	Initialize
$Ntrans$	Number of transmitters in the model	–	Initialize
$\omega$	The weighting parameter in Eq. (15)	–	Initialize
$\alpha$	The weighting parameter in Eq. (18)	–	Initialize
${d}_{r,t}$	Euclidean distance between receiver and transmitter	m	Update among algorithm procedure


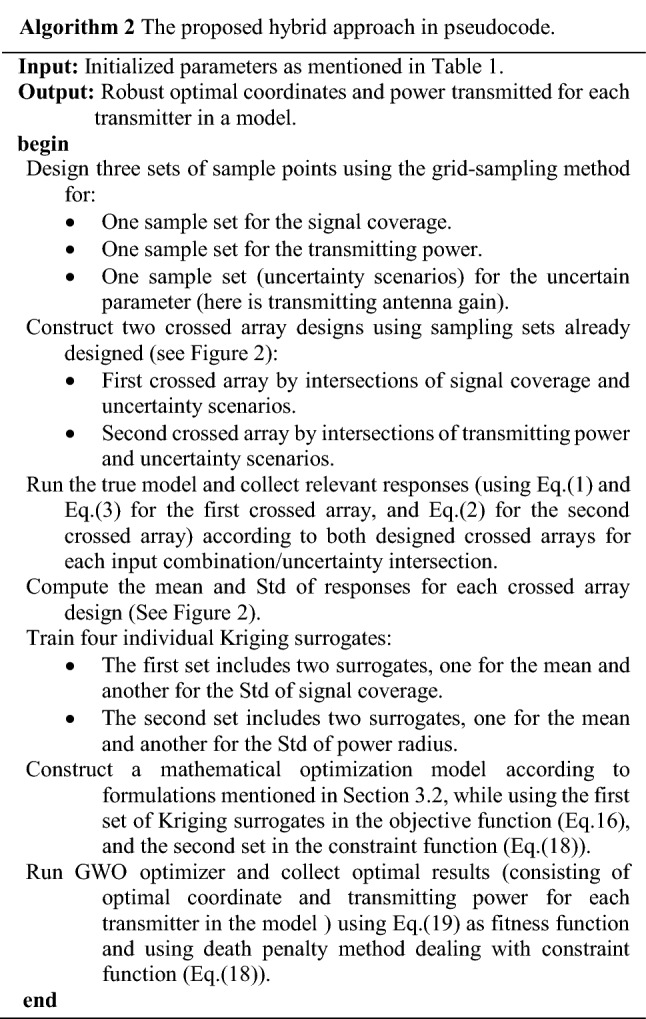
- **Step 2** Design of experiments using crossed array design.
  Design sets of experiments using the structure of crossed array design (see Fig. 2) separately for two different functions, one for power absorbed in Eq. (1) and the second for the power radius in Eq. (2). For each function, also two sets of sample points are designed separately. The first set (over the design variables) is in an inner array and the second set (over an uncertain variable) is in an outer array. The design variables for the absorbed power in Eq. (1) are ${d}_{r,t}$ and ${P}_{t}$, and for power radius in Eq. (2) is ${P}_{t}$. The uncertain variable in both functions is ${\widetilde{G}}_{r}$. Here, we apply the grid-sampling method in the class of space filling design^87^ with resolution ${\Delta \gg} \delta$. In other words, the sampling grid is of considerably lower resolution than $\delta$ to reduce the number of required function evaluations. The information for the regions between the grid points will be obtained through interpolation using the Kriging surrogate.
- **Step 3** Obtain response for each combination of design and uncertain variables.
  Regarding the first crossed sampling design, we execute the power absorbed function using Eq. (1) and accordingly compute the “$\mathrm{Covered}\left(\mathrm{r}\right)$” using Eq. (3) with values 1 or 0. Besides, regarding the second crossed sampling design, we run the power radius function using Eq. (2) and obtain the relevant responses for each combination of design and uncertain parameters in the crossed array designs.
- **Step 4** Compute the statistical measures including mean and Std of responses.
  In this step, the mean and the standard deviation of coverage and power radius are computed for each input combination using Eqs. (12) and (13) regarding a relevant crossed array design. Note that these statistical measures result from the variability of the uncertain parameter that was already considered in the previous steps.
- **Step 5** Construct Kriging surrogates.
  Four Kriging surrogates are constructed separately using the acquired input–output data pairs. The Kriging model is identified for: (i) the mean of the coverage, (ii) the standard deviation of the coverage, (iii) the mean of the power radius, and (iv) the standard deviation of the power radius.
- **Step 6** Validate surrogate models.
  The surrogate model constructed in Step 5 has to be validated to ensure that its predictive power is sufficient for design optimization purposes. Here, validation is executed using the leave-one-out cross-validation ($k=1$)^86,88^, which works as follows. First, delete the ${s}\text{th}$ input combination and the relevant output from the complete set of the $l$ th combination ($s=\mathrm{1,2},\dots ,l$), i.e., to avoid the extrapolation by Kriging, we avoid dropping the sample points in the margin. The model is constructed using $l-1$ remaining rows and predicts the output for the left-out point (${s}_{-1}$). This is realized for all input combinations (sample points) and leads to computing $l$ predictions (${\widehat{y}}_{s}$). Finally, evaluating the standardized residuals are computed as
  20 $${D}_{s}=\frac{{y}_{s}-{\widehat{y}}_{-s}}{\sqrt{\frac{1}{l}\sum_{s=1}^{l}{\left({y}_{s}-{\widehat{y}}_{-s}\right)}^{2}}}$$
  Most of the standardized residuals should be within the interval $-3\le {D}_{s}\le 3$, and any observation outside of this interval (outlier) is potentially unacceptable with regard to its observed simulation output^86,88^.
- **Step 7** Set up the mathematical optimization model.
  The proposed robust optimization model using Eq. (15) through Eq. (19) is arranged.
- **Step 8** Run the GWO optimizer.
  The GWO optimizer is executed using the expression Eq. (19) as a fitness function, and Eq. (18) as a constraint. Here, we control the feasibility of model in constraint using the death penalty for any point out of the feasible region. Note that during the optimization run, all required expressions in both Eqs. (18) and (19) are estimated by the relevant Kriging surrogates constructed in Step 5, so that no further evaluations of the original computational model are required. Another point is that to improve the computational efficiency of the optimization process, we do not investigate all receiver test points $CR\subseteq {\mathbb{Z}}^{2}$ in each iteration. But instead, we consider a smaller set of receiver test points that are randomly selected from the domain.
- **Step 9** Compute the two-sided BCI for an obtained robust optimal point.
  In the stochastic simulation, each input combination $\mathrm{X}$ is replicated several times $\mathrm{m}\ge 1$. In the case of expensive simulations, the number possible of replications is smaller, therefore, parametric bootstrapping is unlikely to produce acceptable results (i.e., it rarely can find the exact distribution of the I/O simulation data)^47,67,89^. Here, to find the bootstrapped set of data, a model is resampled $B$ times $\left(b=\mathrm{1,2},\dots ,B\right)$ (sampling with replacement). Let us assume that ${d}^{+}$ is a robust optimal solution obtained from the procedure in Step 1 through Step 6. It is assumed that ${d}^{+}$ is a robust optimal solution, which is obtained from the original (non-bootstrapped) surrogate. All output values at point ${d}^{+}$ are estimated using all the $B$ bootstrapped surrogates. The distribution-free Bootstrapped Confidence Intervals (BCI) can be computed as below^67,90^:
  21 $$P\left( {d_{\left( {\left\lfloor {B(\gamma /2)} \right\rfloor } \right)}^{ + *} \leqslant {d^ + } \leqslant d_{\left( {\left\lceil {B\left( {1 - \gamma } \right)/2} \right\rceil } \right)}^{ + *}} \right) = 1 - \gamma$$
  where $\gamma /2$ gives two-sided BCI, whereas Bonferroni’s inequality^91^ suggests that the type-I error rate of $\upgamma$^67,92^ for each interval per output is divided by the number of outputs, here, the mean, Std, and the SNR. If the values of the bootstrap estimate ${{d}^{+}}^{*}$ are sorted from low to high, then $\left\lfloor . \right\rfloor$ and $\left\lceil . \right\rceil$, respectively, denotes floor and ceiling function to achieve the integer part and round upwards. Here, it is assumed that the set of sample points is fixed and only old data to fit a surrogate with sufficient replication is available, whereas new simulation replicating is very expensive. This augmented bootstrapping approach does not imply extra computational cost due to resampling, and the required simulation run to find a bootstrapped set of data^72,85,93^. ${x}_{s}$ ($s=\mathrm{1,2},\dots ,l$) denotes the set of sample points and each ${x}_{s}$ is repeated $m$ times ($r=\mathrm{1,2},\dots ,m$). We assume that the original set of data obtained from the original simulation model is available (size $l\times m$) when $m$ is the number of scenarios for uncertainty and $l$ is the number of input combinations. Moreover, the augmented bootstrapping procedure is sketched in Algorithm 3.

## Experimental benchmark problems

### The algorithm setup

In this section, three cases featuring a different number of transmitting antennas (e.g., two, three, and four base stations) are considered to evaluate the performance of the proposed algorithm for reduced cost transmitting antenna placement under uncertainty. Following we also compare the effectiveness of the proposed hybrid approach compare with standalone GWO (uncombined with a surrogate) and the Non-dominated Sorting Genetic Algorithm (NSGAII)^94^ in cases with a higher number of transmitters (up to 20 base stations). The comparison results are provided in terms of accuracy (higher coverage rate and higher power transmitted), robustness (reliability due to uncertainty using SNR criterion), and computational cost (number of required function evaluations and total time elapsed for computing). The transmitters are assumed to be homogeneous (i.e., transmitters are of only one type and have the same cost). In each case, the optimization model simultaneously considers the coverage and transmit power as objective functions, and the overlap as a constraint. The free-space propagation model is used to measure the signal strength in each case. The initial parameters used in the proposed algorithm are adjusted as follows. The two-dimensional map size is $120\times 120 \,{\mathrm{m}}^{2}$, and the grid resolution $\delta$ of the map is $10 \,\mathrm{m}$. All radiation patterns of the transmitters are assumed to be omnidirectional. The receiving antenna gain ${G}_{r}$ is $1 \,\mathrm{mW}$, and wavelength $\lambda$ is $0.125\, \mathrm{m}$. The ${\theta }_{r}$ threshold for all receivers is $1\times {10}^{-7}$ $\mathrm{mW}$.
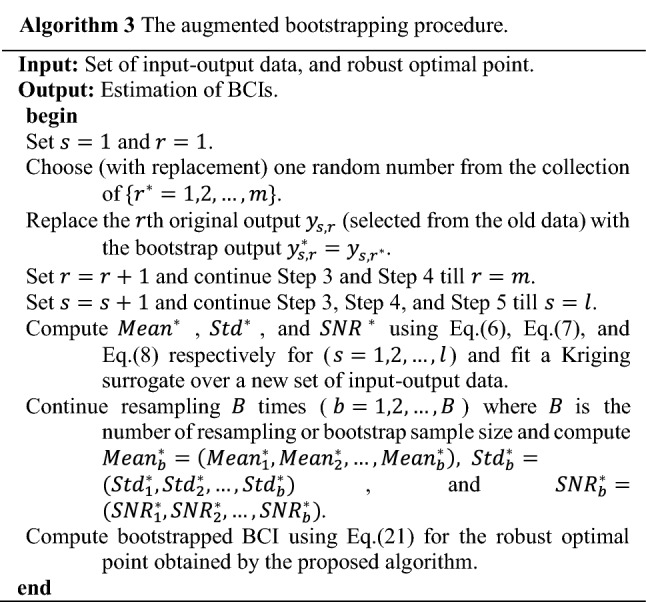


The mathematical model for each test case (i.e., with two, three, or four transmitters) is constructed according to equations Eq. (15) through Eq. (19). In each case, the design variables are the coordinate ($${x}_{t},{y}_{t})$$ and transmitting power $${P}_{t}$$ of each transmitter, and the transmitting antenna gain $${\widetilde{G}}_{t}$$ is assumed to be an uncertain parameter. The design ranges for design variables are $${0\le x}_{t}\le 120$$, $${0\le y}_{t}\le 120$$, and $${0.5\le P}_{t}\le 2.5$$, ($${P}_{max}=2.5$$ used in Eq. (17). The uncertain parameter $${\widetilde{G}}_{t}$$ is also assumed to vary uniformly in the range 0.5 to 1.5. In the optimization procedure, the Std of the coverage is weighted three times of mean by considering $$\omega =3$$ in Eq. (16). We allow 30 percent average intersection between all signals transmitted by multiple transmitters in a model, thus $$\beta =0.3$$ considered in Eq. (18). Additionally, to compare the results in all cases, the parameter $$\alpha$$ in Eq. (19) is set to zero. Furthermore, the sensitivity analysis is conducted with different values of $$\alpha$$ in $$\left[-2,+2\right]$$, separately for each case. We apply $$50\times 30$$ samples (50 input combinations with 30 uncertainty scenarios) regarding the crossed array for coverage functions (Eq. (1) and Eq. (3)) and fit Kriging surrogates for the mean and Std of coverage. Also, $$50\times 30$$ samples are employed involving the crossed array design for computing the mean and Std of power radius using Eq. (2) and fitting two relevant Kriging surrogates. To evaluate the reliability of obtained results, the optimization procedure is repeated 10 times for each case. We employ the Matlab® environment for data and function analysis. The DACE^60^, Matlab® toolbox has been employed to construct the Kriging surrogate with zero-order polynomial regression and Gaussian correlation functions. The Matlab® function “*gridsamp”* in the DACE toolbox is used for sampling design for both design parameters (inner array) and uncertain parameters (outer array) according to crossed array configuration (see Fig. 2).

### Robust optimal positioning

**Figure 4:**
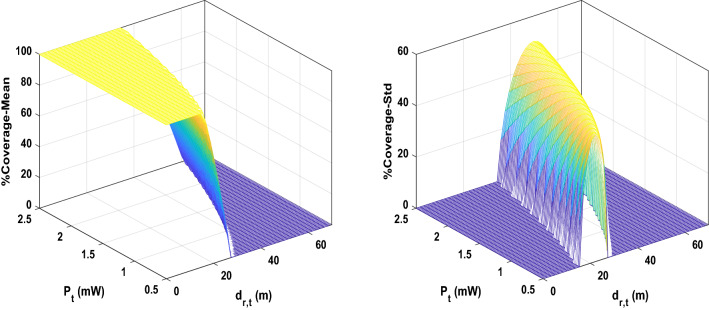
The 3D surface plot for 2,500 input combinations in crossed array design.

**Figure 5:**
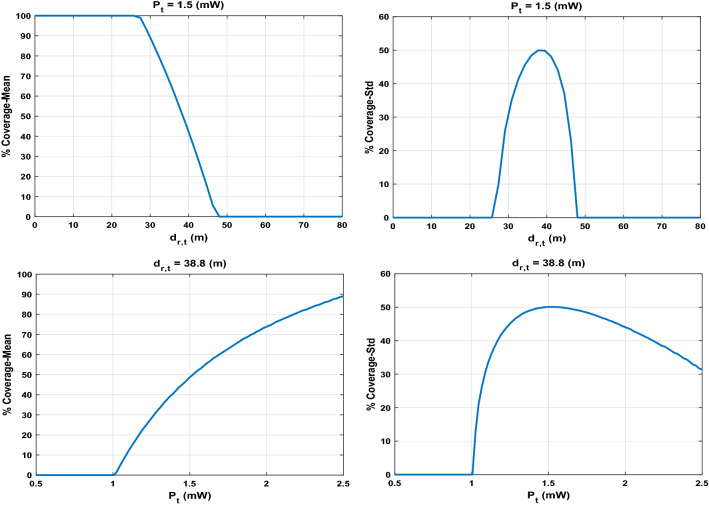
The mean and Std of coverage over each input parameter in an inner array of crossed design. In each figure, the second parameter fixed in certain value, ${P}_{t}=1.5 (\mathrm{mW})$ and ${d}_{r,t}=38.8 (\mathrm{m})$.

**Figure 6:**
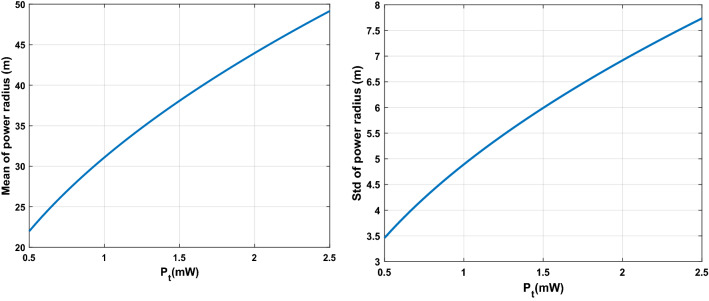
The mean and Std of power radius $(\mathrm{m})$ over the power transmitted $(\mathrm{mW})$ in $[-3,+3]$.

**Figure 7:**
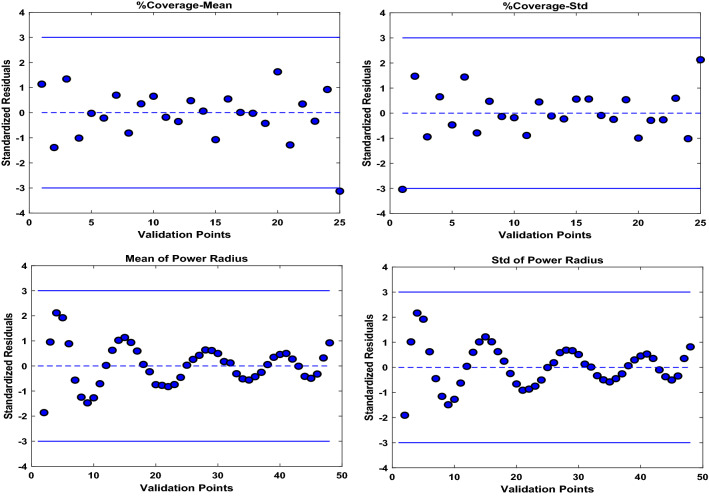
Scatterplots for the cross-validation of the Kriging surrogates. Shown are the standardized residuals in the range of $[-\mathrm{3,3}]$ for the mean and Std of coverage and power radius.

For each case of $Ntrans=2, 3$, and $4$ transmitters, we run the algorithm ten times to compute the relevant statistical measures to account for the randomness in the proposed stochastic optimization algorithm. Figure 4 shows the 3D surface plot for mean and Std of coverage over input combinations in the crossed array (${d}_{r,t}$ and ${P}_{t}$). As it can be seen, there is a nonlinear relationship between the input and output set of data in the crossed array design because of the existence of uncertainty in the model. Figure 5 illustrates the mean and Std of coverage over one input parameter (${d}_{r,t}$ or ${P}_{t}$) while the other is fixed at a predefined value. The power radius function of Eq. (2) is applied to collect the data regarding each crossed design and uncertain parameters. Figure 6 shows the mean and Std of power radius ($\mathrm{m}$) overpower transmitted ($\mathrm{mW}$). Using the input–output data pairs obtained from the crossed array, two Kriging surrogates are fitted, one for the mean and the other for the Std of the coverage. Two other Kriging surrogates are fitted using the input–output data pairs in the relevant crossed array design, one for the mean of power radius and another one for Std of power radius. The prediction errors for all four Kriging surrogates are computed by the leave-one-out cross-validation approach. As shown in Fig. 7, most observed standardized residuals are within the interval $\left[-\mathrm{3,3}\right]$, which ensures sufficient accuracy of the surrogates.

**Figure 8:**
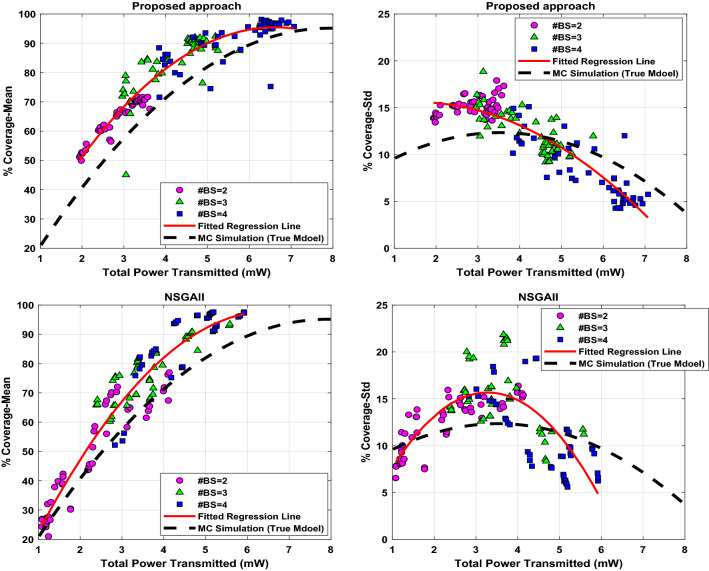
The estimation of the Pareto front using the proposed approach and NSGAII method for the mean and standard deviation of the coverage versus the transmitted total power. The estimation is obtained regarding different values of the weighting parameter $\mathrm{\alpha }$.

In the next step, the robust optimization model is constructed using Eq. (19) as an objective function and Eq. (18) as the constraint. In both functions, we employ the Kriging surrogates that are already identified for the mean and the standard deviation of the coverage and the power radius. Accordingly, the GWO is run to obtain the robust optimal solution (${x}_{t}, {y}_{t}$, and ${P}_{t}$) for each case (e.g., the cases with number of two, three, or four transmitters). As mentioned earlier, optimization runs are repeated 10 times for each case. This optimization procedure is performed for different values of the parameter $\alpha$ in Eq. (19) to investigate the effects of $\alpha$ on the robust optimal results. Toward this end, the parameter $\alpha$ is changed from − 2 to + 2 with a step size of 1. The results obtained by the proposed hybrid approach for all three cases of two, three and four transmitters. Table 2 gathers the obtained statistical results. Using all collected data provided in Table 2 for the proposed hybrid approach by varying threshold $\alpha$ in Eq. (19), the Pareto-optimal efficiency frontier is estimated, where we consider the mean and Std as criteria between which finding a trade-off. Figure 8 illustrates the mean and the Std versus the total transmitted power using all samples that were already collected during the previous optimization runs. This figure also provided the Pareto front estimation using the common NSGAII method known for such multi-objective problems. As can be seen, the results are competitive, and given these results, the decision-makers select their preferred combination of the mean and Std regarding the power consumed to transmit the signals in a predefined network zone.

**Table 2:** Statistical results over 10 repetitions of proposed Kriging-GWO (hybrid surrogate-metaheuristic) approach in robust optimal placement of two, three, and four transmitting antennas for different "$\alpha$" values in Eq. (19).

$\alpha$	%Coverage_Mean				%Coverage_Std				SNR				TPT (mW)			
	Avg	Std	Max	Min	Avg	Std	Max	Min	Avg	Std	Max	Min	Avg	Std	Max	Min
$$\#{\varvec{B}}{\varvec{S}}=2$$																
− 2	52.47	1.41	49.95	55.66	14.17	0.45	13.44	15.28	− 5.68	0.22	− 6.09	− 5.18	2.01	0.05	1.94	2.10
− 1	60.16	1.81	56.47	62.15	15.41	0.59	14.51	16.53	− 4.53	0.27	− 5.08	− 4.25	2.53	0.10	2.39	2.69
0	68.05	2.17	65.04	70.32	14.91	0.69	13.70	16.23	− 3.48	0.28	− 3.87	− 3.18	3.07	0.18	2.82	3.32
+ 1	69.32	1.68	66.43	71.60	15.39	1.24	13.64	17.90	− 3.33	0.21	− 3.68	− 3.03	3.26	0.20	2.83	3.50
+ 2	69.52	1.90	66.16	71.65	15.81	0.83	14.52	17.33	− 3.32	0.24	− 3.74	− 3.07	3.34	0.21	2.91	3.62
$$\#{\varvec{B}}{\varvec{S}}=3$$																
− 2	69.93	9.00	45.05	78.96	14.94	1.86	11.96	18.86	− 3.34	1.28	− 6.96	− 2.20	3.07	0.08	2.96	3.23
− 1	82.28	2.46	77.49	84.62	14.04	0.81	12.30	15.29	− 1.87	0.26	− 2.38	− 1.61	3.76	0.19	3.42	4.06
0	88.77	2.32	83.26	91.38	11.28	1.25	9.87	13.75	− 1.17	0.24	− 1.75	− 0.89	4.71	0.17	4.39	4.98
+ 1	89.52	4.42	76.39	91.86	11.21	1.19	9.60	13.92	− 1.10	0.47	− 2.48	− 0.84	4.73	0.14	4.52	4.91
+ 2	90.09	1.73	87.42	92.41	10.09	0.83	9.20	11.97	− 1.01	0.17	− 1.31	− 0.79	4.95	0.37	4.53	5.76
$$\#{\varvec{B}}{\varvec{S}}=4$$																
− 2	82.87	4.64	71.59	88.45	12.47	1.64	10.14	15.11	− 1.78	0.52	− 3.05	− 1.17	4.03	0.15	3.84	4.34
− 1	88.78	5.49	74.53	93.60	9.35	1.82	7.24	13.02	− 1.14	0.59	− 2.67	− 0.63	5.08	0.22	4.65	5.37
0	92.40	6.31	75.24	97.83	7.46	2.22	4.25	12.01	− 0.77	0.66	− 2.58	− 0.21	6.18	0.28	5.65	6.55
+ 1	96.60	1.10	93.98	98.15	5.06	0.56	4.27	5.97	− 0.33	0.10	− 0.58	− 0.19	6.47	0.19	6.26	6.94
+ 2	96.48	0.89	94.95	97.90	5.42	0.67	4.59	6.95	− 0.35	0.09	− 0.49	− 0.21	6.67	0.20	6.36	7.07

To study more the obtained results in Table 2 by serving examples, for case $\alpha =0$ , the solution with the highest SNR among all 10 repetitions are selected as a robust optimal point. Table 3 shows the best results (corresponding to the higher SNR value) for the placement problems with two, three, and four transmitters. Figure 9 shows the coverage using the robust optimal results depicted in Table 3 for the cases with two, three, and four transmitters separately. The plots are shown for the mean of the power radius, $mean+3Std$, and $mean-3Std$*.* The results indicate that the mean of the coverage is $91.38\%$ (with $\mathrm{Std}=10.01\mathrm{\%}$) by using $\mathrm{TPT}=4.56 \left(\mathrm{mW}\right)$ is obtained in the case of three transmitters. For the problem with two transmitters, the total transmitted power is $3.18 \left(\mathrm{mW}\right)$ to provide $70.30\%$ of the mean coverage with $\mathrm{Std}=13.70\mathrm{\%}$. Finally, for the problem with four transmitters, the mean coverage of $97.83\%$ can be obtained with $\mathrm{Std}=4.25\mathrm{\%}$, when the total power is $6.41 \left(\mathrm{mW}\right)$. Note that four transmitters ensure higher SNR, but it also leads to a higher average overlap. The average intersection of transmitters is kept at less than $\beta =30\%$ due to the predefined model’s constraint in Eq. (18). In general, the three transmitters case seems to be the most advantageous from the point of view of ensuring the best trade-off between the coverage, robustness, power consumption, and overlap.

**Table 3:** The optimal results with the highest SNR among 10 repetitions of proposed algorithm for placement of two, three, four transmitting antennas ($\beta =0.3, \alpha =0$).

#BS	Optimal Location								Power Transmitted (mW)					% Coverage		
	${x}_{t1}$	${y}_{t1}$	${x}_{t2}$	${y}_{t2}$	${x}_{t3}$	${y}_{t3}$	${x}_{t4}$	${y}_{t4}$	${P}_{t1}$	${P}_{t2}$	${P}_{t3}$	${P}_{t4}$	Sum	Mean	Std	SNR
#2	30	30	90	91	–	–	–	–	1.55	1.63	–	–	3.18	70.30	13.70	− 3.18
#3	99	18	17	45	72	99	–	–	1.63	1.54	1.40	–	4.56	91.38	10.01	− 0.89
#4	14	17	90	69	92	50	8	107	1.62	1.70	1.75	1.35	6.41	97.83	4.25	− 0.21

**Figure 9:**
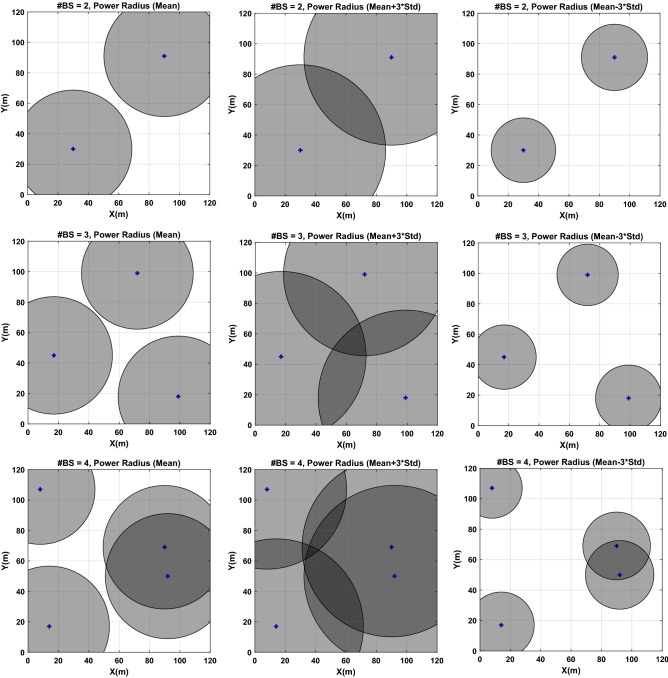
2D Visualization of the robust optimal coverage for the problems with placement of two, three, and four transmitters. Shown are the mean, $mean+3Std$, and $mean-3Std$.

### Bootstrapping (sensitivity analysis)

Here, instead of estimating a single robust optimal point using a particular response that might be inaccurate because of high variability in uncertain parameters, we derive a series of possible responses that consider the degree of uncertainty by providing confidence regions or prediction intervals. This is realized by resampling adopted to the uncertain component. In this study, we set the bootstrapped sample size $B=100$, and $\gamma =0.05$. For each bootstrapped sample, we randomly select 30 uncertainty scenarios from the original sample points that were already available in the crossed array design (with the same sample size of 30). Subsequently, regarding the new sample sets, the mean and the standard deviation of the coverage are computed for the obtained robust optimal points as shown in Table 3. However, 95% two-sided approximations of BCI obtained by the distribution-free bootstrapping technique for the mean and the standard deviation of the coverage in the robust optimal points and the relevant SNR are as follows:

$$\left\{ \begin{aligned} & P\left( {Mean(d^{ + } )^{*}_{{\,\left( {\left\lfloor{100(0.05/2)}\right\rfloor } \right)}} \le Mean(d^{ + } ) \le Mean(d^{ + } )^{*}_{{\left({\left\lceil {100(1 - (0.05/2)}\right\rceil } \right)}} } \right) = 0.95 \hfill \\& P\left( {Std(d^{ + } )^{*}_{{\left( {\left\lfloor{100(0.05/2)}\right\rfloor } \right)}} \le Std(d^{ + } ) \le Std(d^{ + } )^{*}_{{\left( {\left\lceil {100(1 - (0.05/2)}\right\rceil } \right)}} } \right) = 0.95 \hfill \\& P\left( {SNR(d^{ + } )^{*}_{{\left( {\left\lfloor {100(0.05/2)}\right\rfloor } \right)}} \le SNR(d^{ + } ) \le SNR(d^{ + } )^{*}_{{\left({\left\lceil {100(1 - (0.05/2)} \right\rceil }\right)}} } \right) = 0.95 \hfill \\ \end{aligned} \right.$$

$$\begin{aligned} \# {\text{BS}} = 02:{ }\left\{ {\begin{array}{*{20}c} {68.30 \le Mean(d^{ + } ) \le 72.22} \\ {14.97 \le Std(d^{ + } ) \le 16.93} \\ { - 3.47 \le SNR(d^{ + } ) \le - 2.99} \\ \end{array} } \right. \hfill \\ \# {\text{BS}} = 03:{ }\left\{ {\begin{array}{*{20}c} {83.33 \le Mean(d^{ + } ) \le 86.86} \\ {14.07 \le Std(d^{ + } ) \le 16.30} \\ { - 1.80 \le SNR(d^{ + } ) \le - 1.42} \\ \end{array} } \right. \hfill \\ \# {\text{BS}} = 04:{ }\left\{ {\begin{array}{*{20}c} {94.66 \le Mean(d^{ + } ) \le 96.31} \\ {6.47 \le Std(d^{ + } ) \le 8.06} \\ { - 0.56 \le SNR(d^{ + } ) \le - 0.38} \\ \end{array} } \right. \hfill \\ \end{aligned}$$

**Figure 10:**
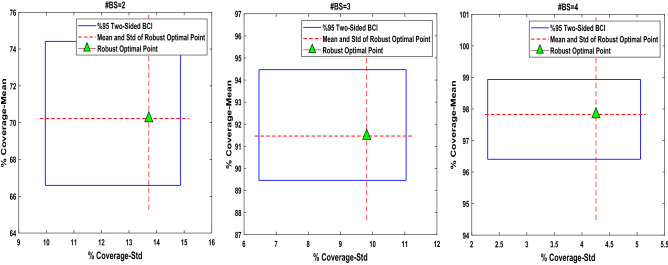
The 95% two-sided confidence region obtained by free-distribution bootstrapping technique for placement problems with two, three, and four transmitters.

Figure 10 illustrates the 95% confidence regions for the mean and the standard deviation of the coverage at the original robust optimal points (as shown in Table 3).

**Figure 11:**
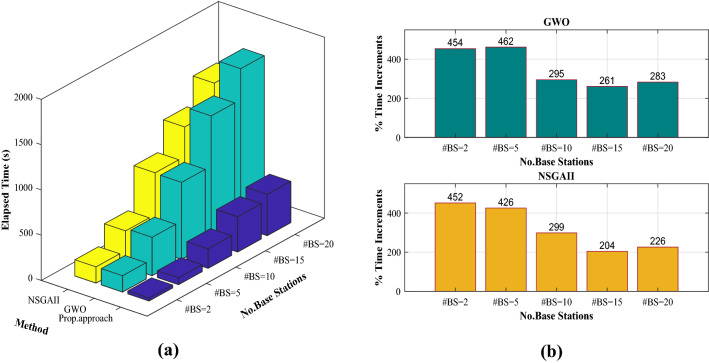
The total computational time consuming (10 separate repetitions) for robust optimal positioning of different sizes of base statements (number of transmitters), (**a**) time elapsed using each method, (**b**) percent increment of time elapsing for standalone GWO and NSGAII compare with proposed hybrid Kriging/GWO approach.

### Comparative study

Here, a deep comparison between the proposed hybrid surrogate/metaheuristic approach (Kriging/GWO) with standalone metaheuristic (GWO) and NSGAII method are provided for robust optimal placements of multiple transmitters problem. Results are compared in terms of accuracy (higher coverage rate and higher power transmitted), robustness (reliability against uncertainty with SNR criterion), and computational cost (number of required function evaluations and total computation time consuming). We employ all three methods using different sizes of transmitters. The model’s parameters including $\beta$ in Eq. (18) and $\alpha$ in Eq. (19) are adjusted to $0.5$ and 0 respectively, and other parameters are tuned the same as what is mentioned in “The algorithm setup” section. All methods are executed 10 times separately for each size of transmitters (number of base stations) to study the effect of randomness as well. As mentioned in “The algorithm setup” section, in our proposed algorithm, we apply $50\times 30$ samples to construct the crossed array for the coverage (Eqs. (1) and 3) using to fit Kriging surrogates for the mean and Std of coverage. In addition, $50\times 30$ samples are employed in the crossed array design to compute the mean and Std of power radius using Eq. (2) and training two relevant Kriging surrogates. To speed up the optimization procedure, we set grid resolution $\delta$ to 10 m, so instead of evaluation all possible receiver test points in network $\left(120\times 120\right)$, we evaluate $12\times 12$ test points in each fitness evaluation by optimizer. The GWO optimizer which is combined with Kriging surrogates is derived to obtain the robust optimal solution for the mathematical optimization model described in “Mathematical optimization model” section. Note that the number of optimizer’s runs can be chosen large enough without worrying about the computational cost because the Kriging surrogates are used to predict the responses instead of the true model in each optimizer’ run. However, if the optimizer, e.g., GWO is used individually (not combined with a surrogate), let's compute the required number of function evaluations by considering 10 search agents with 20 maximum iterations, and 30 uncertainty scenarios to obtain statistical measures of mean and Std. The total number of fitness evaluations required for the standalone GWO optimizer (see “Computational cost” section) is equal to $\left[10\times 20\times 30\times \left(12\times 12\right)\times No.Transmitetrs\right]$. It clear that by increasing the number of transmitters in the model, the required number of function evaluations in each optimizer’s run is mounted significantly. To be fair comparison, the NSGAII also is adjusted to employ the same number of function evaluations. All three methods are derived for robust coverage optimization of cases with 2, 5, 10, 15, and 20 transmitters. The obtained statistical results for all three approaches are given in Table 4. As it can be seen from the obtained results, in terms of accuracy (higher coverage rate and higher power transmitted) and robustness against uncertainty (greater SNR), the proposed Kriging/GWO (hybrid surrogate-metaheuristic) approach outperforms the GWO (uncombined metaheuristic) for cases with 5, 10, 15, and 20 number of transmitters (base stations). The GWO provides better performance in accuracy and robustness for the case with two base stations. Besides, the proposed hybrid approach outperforms NSGAII in cases with the sizes of 2 and 5 transmitters and provides competitive performance (accuracy and robustness) with NSGAII for problems with 10, 15, and 20 base stations. While, in a term of computational time consumption, as shown in Fig. 11, both GWO and NSGAII approaches significantly elapsed much more computational time compared with the proposed approach. The average computational time consumed for robust optimal transmitters’ placements using the proposed approach is 350% and 320% less than the time elapsed using GWO and NSGAII respectively. Notably, the total time elapsed by the proposed hybrid approach is counted to include all algorithmic steps consisting of the sampling design for crossed arrays, collecting data, constructing surrogates, and running an optimizer to obtain optimal results. Consequently, the proposed less-expensive approach by integrating surrogate and metaheuristic (Kriging/GWO in this study) can be derived using more cheaply procedure both in terms of a number of required function evaluations and computational time consuming to effectively obtain the robust optimal placements of multiple transmitting antennas in coverage optimization problems under uncertainty. Here, it is worth mentioning that the computational time issue is more emphasized for real-time coverage analysis and optimization of such WSN problems in digital twins or cyber-physical systems, see^95,96^.

**Table 4:** Statistical results over 10 repetitions of the proposed Kriging-GWO (hybrid surrogate-metaheuristic) approach compare to GWO (uncombined metaheuristic) and NSGAII in robust optimal placement over the different size of base stations (transmitting antennas), while $\beta =0.5$ in Eq. (18), and $\alpha =0$ in Eq. (19).

$\#BS$	%Coverage_Mean				%Coverage_Std				SNR				TPT (mW)			
	Avg	Std	Max	Min	Avg	Std	Max	Min	Avg	Std	Max	Min	Avg	Std	Max	Min
**Proposed hybrid Kriging/GWO (surrogate/metaheuristic) approach**																
2	75.53	6.16	62.71	87.66	16.37	0.62	15.40	17.68	− 2.66	0.70	− 4.19	− 1.38	3.13	0.71	2.14	4.99
5	98.71	1.12	95.65	99.72	2.51	1.41	0.96	6.04	− 0.12	0.11	− 0.43	− 0.03	6.63	0.51	5.84	7.29
10	99.47	1.45	95.14	100.00	0.90	2.32	0.00	7.82	− 0.05	0.15	− 0.50	0.00	11.78	0.83	10.53	13.32
15	99.96	0.07	99.82	100.00	0.11	0.22	0.00	0.55	0.00	0.01	− 0.02	0.00	15.67	2.17	11.61	18.34
20	99.99	0.03	99.91	100.00	0.03	0.09	0.00	0.30	0.00	0.00	− 0.01	0.00	20.94	4.81	11.47	27.15
**GWO (uncombined metaheuristic)**																
2	78.85	2.30	75.05	82.55	14.57	1.42	12.58	16.91	− 2.24	0.24	− 2.61	− 1.85	3.49	0.31	3.00	3.87
5	98.44	0.79	97.27	99.56	3.35	1.10	1.44	5.03	− 0.15	0.08	− 0.27	− 0.04	4.83	0.34	4.38	5.32
10	96.44	10.45	65.09	100.00	1.58	3.84	0.00	13.04	− 0.39	1.14	− 3.82	0.00	6.32	0.53	5.58	7.32
15	95.18	5.32	88.77	100.00	6.54	7.20	0.00	15.20	− 0.54	0.60	− 1.27	0.00	9.48	0.89	8.24	11.47
20	95.87	4.74	87.25	100.00	4.47	4.23	0.00	10.40	− 0.42	0.48	− 1.29	0.00	13.08	2.12	11.08	17.46
**NSGAII**																
2	61.59	12.23	42.62	78.89	14.35	1.09	12.08	15.84	− 4.49	1.71	− 7.44	− 2.23	2.72	0.79	1.62	3.83
5	95.28	6.12	78.15	99.95	5.44	4.40	0.25	16.09	− 0.49	0.66	− 2.34	0.00	5.80	0.85	4.13	7.03
10	99.95	0.09	99.72	100.00	0.14	0.30	0.00	0.87	0.00	0.01	− 0.03	0.00	12.25	0.74	11.14	13.52
15	100.00	0.00	100.00	100.00	0.00	0.00	0.00	0.00	0.00	0.00	0.00	0.00	18.61	1.51	16.53	21.40
20	100.00	0.00	100.00	100.00	0.00	0.00	0.00	0.00	0.00	0.00	0.00	0.00	26.40	3.55	21.05	34.91

## Conclusion

This paper presented a novel algorithm for solving the homogeneous transmitter placement problem under uncertainty. Our methodology employs a hybrid Kriging-GWO approach combined with robust design optimization. The proposed algorithm enables computing the statistical measures due to the source of variability (uncertainty) and evaluating the possible positions and receiver test points to obtain robust optimal placement of multiple transmitters at a low computational cost. Multiple objectives including the mean and the Std of the coverage, the total required transmitted power, as well as the reliability of the model by controlling the overlap, are all considered simultaneously. The proposed approach is applied effectively for robust coverage optimization with two, three, and four transmitters. The comparative study is conducted to evaluate the performance of the proposed approach with two common optimizers of GWO (when applied standalone) and NSGAII for cases with different sizes of transmitting, e.g., 2, 5, 10, 15, and 20 in the model in terms of accuracy, robustness, and computational time consumption. The results confirm the effectiveness of the less-expensive proposed hybrid approach by integrating Kriging and GWO to obtain the robust optimal placement of transmitting antennas using much less time elapsed for computational procedure compared with standalone optimizers.

However, this study limits itself in some points that future research can be devoted to overcoming those limitations of the current study as follows. Other surrogates such as ANN, RBF, polynomial regression, and polynomial chaos expansion can be combined with also some other common metaheuristics such as NSGAII, PSO, ACO, and GA. Instead of conventional robust dual surface, recent developments in robust optimization approaches may be served to tackle the existence of uncertainty in the model, see^39,40,97,98^. The proposed approach can manage to consider other uncertainty distributions e.g., Gaussian among the crossed array framework in addition of uniform distribution in this paper, see^72^ for such cases with an unknown probability distribution of uncertainty. The optimization problem for the transmitter placement problem can be expanded to obtain the required number of transmitters as a new design variable. In addition, other objective functions such as transmitters cost, or demand rate also can be attended to in an optimization model. Instead of transmitting antenna gains, uncertainty in some other physical parameters can be considered as well.

## Supplementary Information

Supplementary Information.

## Data Availability

All data generated or analyzed during this study are included in this published article and the results would be reproducible using the supplementary information files that are shared on the link below: Supplementary Information.
