# Author Correction: A data driven approach in less expensive robust transmitting coverage and power optimization

**DOI:** 10.1038/s41598-022-26360-2

**Published:** 2022-12-22

**Authors:** Amir Parnianifard, Shahid Mumtaz, Sushank Chaudhary, Muhammad Ali Imran, Lunchakorn Wuttisittikulkij

**Affiliations:** 1 Department of Electrical Engineering, Faculty of Engineering, Chulalongkorn University, Bangkok, 10330 Thailand; 2 Instituto de Telecomunicações, Campus Universitário de Santiago, 3810-193 Aveiro, Portugal; 3 School of Engineering, University of Glasgow, Glasgow, G12 8QQ UK; 4 School of Engineering, Nottingham Trent University, Nottingham, UK

Correction to: *Scientific Reports* 10.1038/s41598-022-21490-z, published online 22 October 2022

The original version of this Article omitted an affiliation for Shahid Mumtaz. The correct affiliations are listed below.

Instituto de Telecomunicações, Campus Universitário de Santiago, 3810‑193 Aveiro, Portugal.

School of Engineering, Nottingham Trent University, Nottingham, UK.

The original Article has been corrected.

